# SARS-CoV-2 PrEP complicates antibody testing after vaccination: a call for awareness

**DOI:** 10.1007/s00277-022-04859-y

**Published:** 2022-05-24

**Authors:** Malte B. Monin, Benjamin Marx, Lisa Meffert, Christoph Boesecke, Jürgen K. Rockstroh, Christian P. Strassburg, Peter Brossart, Annkristin Heine

**Affiliations:** 1 Department of Internal Medicine I, University Hospital Bonn, Venusberg-Campus 1 53127, Bonn, Germany; 2 German Centre for Infection Research (DZIF), Partner-site Cologne-Bonn, Bonn, Germany; 3 Institute of Virology, Faculty of Medicine, University of Bonn, Bonn, Germany; 4 Department of Internal Medicine III, University Hospital Bonn, Bonn, Germany

Dear Editor,

Patients with cancer, especially with hematological malignancies, were encouraged to be vaccinated early for SARS-CoV-2. Yet, these patients with dysfunctional or depleted B-lymphocytes frequently fail to reach positive SARS-CoV-2 anti-spike IgG titers after vaccination. To prevent severe COVID-19, combined monoclonal neutralizing antibodies (Ronapreve®: casirivimab/imdevimab; Evusheld®: tixagevimab/cilgavimab) were approved as pre-exposition prophylaxis (PreP) for these patients [1, 2]. Since several studies have suggested that additional booster vaccinations might elicit protective B and T cell responses in this vulnerable patient cohort, extra booster vaccinations have been recommended [3].

We report here on three CD20-depleted lymphoma patients, showing negative SARS-CoV-2 anti-spike IgG after two vaccinations. Thus, patients were treated with casirivimab/imdevimab infusions that were recommended to be applied monthly. Due to the development of novel virus variants and its ineffectiveness against BA.1, we stopped the treatment. When the patients were tested for SARS-CoV-2 anti-spike IgG 12 weeks after the last infusion and four weeks after booster vaccination, we detected extremely high titers of SARS-CoV-2 anti-spike IgG (all ≥ 5680 binding antibody units per milliliter). These IgGs were most likely not induced by active vaccination, but rather by the application of casirivimab/imdevimab. This finding suggests that passive immunization with the monoclonal antibodies casirivimab/imdevimab results in antibody titers that are detectable in the blood much longer than one month, which coincides with the described half-life [1]. Of note, in one case, neither SARS-CoV2 nucleocapsid IgA nor SARS-CoV2 nucleocapsid IgG could be detected despite SARS-CoV-2 infection after vaccination and after PrEP therapy indicating that patient’s B-lymphocytes were still reduced without the capability of mounting an IgG response (Table 1). In line, we found an absolute B-lymphocyte depletion in this patient (0.0% CD19^+^/CD20^+^-positive cells).

**Table 1:** SARS-CoV-2 antibody titers

	Sex	Vaccine	SARS-CoV-2 anti-spike IgG [BAU/mL] - After full vaccination, prior PrEP	SARS-CoV-2 anti-spike IgG [BAU/mL] - 12 weeks after PrEP	SARS-CoV-2 surrogate neutralization antibodies [%] - 12 weeks after PrEP	SARS-CoV-2 nucleocapsid IgA/IgG
Patient 1	Male	3 × BNT162b2 by Pfizer BioNTech	Negative	≥ 5680	99.4	Negative despite infection with SARS-CoV-2
Patient 2	Male	3 × BNT162b2 by Pfizer BioNTech	Negative	≥ 5680	99.6	No infection with SARS-CoV-2
Patient 3	Female	2 × BNT162b2 by Pfizer BioNTech 1 × x mRNA-1273 by Moderna	Negative	≥ 5680	99.4	No infection with SARS-COV-2

SARS-CoV-2 IgG II Quant chemiluminescent microparticle immunoassay (Abbott Laboratories) was used according to manufacturer’s instructions to quantify IgG antibodies against SARS-CoV-2 spike receptor-binding domain (SARS-CoV-2 anti-spike IgG). To identify the portion of SARS-CoV-2 surrogate neutralization antibodies in relation to all antibodies [%], a blocking ELISA detection tool (cPass™ SARS-CoV-2 Neutralization Antibody Detection Kit; GenScript) was used. Elecsys Anti SARSCoV-2 chemiluminescent immunoassay (Roche) was used for qualitative detection of SARS-CoV-2 anti-nucleocapsid IgG. Subjects having undergone an infection with SARS-CoV-2 are positive for SARS-CoV-2 anti-nucleocapsid IgG. After vaccination without a prior infection with SARS-CoV-2, SARS-CoV-2 anti-nucleocapsid IgG is negative

*Abbreviations*: *BAU/ml* binding antibody units per milliliter, *PrEP* pre-exposition prophylaxis

The fact that SARS-CoV-2 PrEP causes positive SARS-CoV-2 anti-spike IgG titers over weeks — especially with high proportion of surrogate neutralization antibodies — will complicate to define response rates after extra booster vaccinations in patients with dysfunctional B-lymphocytes. We recommend to take this information into consideration, as new combined monoclonal antibodies are currently available for SARS-CoV-2 PrEP that are highly effective against BA.1, for example tixagevimab/cilgavimab has to be administered only once every six months.

In summary, (1) testing for SARS-CoV-2 anti-spike IgG titers after extra booster vaccinations is challenging in patients having received monoclonal SARS-CoV2 antibodies and (2) monoclonal antibody treatment has to be taken into account when testing for titers is performed. In case of new vaccines being effective against BA.1 and BA.2, only measurements of live-cell neutralization antibodies would help to discriminate between PrEP and vaccination response rates — which would not be eligible for standard diagnostics due to the complex analysis.
